# Novel Utilization of Therapeutic Coatings Based on Infiltrated Encapsulated Rose Bengal Microspheres in Porous Titanium for Implant Applications

**DOI:** 10.3390/pharmaceutics14061244

**Published:** 2022-06-12

**Authors:** Francesca Accioni, Giovanna Rassu, Belén Begines, Luisa Marleny Rodríguez-Albelo, Yadir Torres, Ana Alcudia, Elisabetta Gavini

**Affiliations:** 1 Departmento de Química Orgánica y Farmacéutica, Facultad de Farmacia, Universidad de Sevilla, 41012 Sevilla, Spain; francesca.accioni@gmail.com (F.A.); bbegines@us.es (B.B.); 2 Department of Medical, Surgical and Experimental Sciences, University of Sassari, 07100 Sassari, Italy; eligav@uniss.it; 3 Departmento de Ingeniería y Ciencia de los Materiales y del Transporte, Escuela Politécnica Superior, Universidad de Sevilla, 41004 Sevilla, Spain; iralbelo@us.es (L.M.R.-A.); ytorres@us.es (Y.T.)

**Keywords:** biomaterials, porous titanium, implants, rose bengal, microspheres, drug delivery, polymers, bioactivity, antimicrobial activity

## Abstract

Despite the increasing progress achieved in the last 20 years in both the fabrication of porous dental implants and the development of new biopolymers for targeting drug therapy, there are important issues such as bone resorption, poor osseointegration, and bacterial infections that remain as critical challenges to avoid clinical failure problems. In this work, we present a novel microtechnology based on polycaprolactone microspheres that can adhere to porous titanium implant models obtained by the spacer holder technique to allow a custom biomechanical and biofunctional balance. For this purpose, a double emulsion solvent evaporation technique was successfully employed for the fabrication of the microparticles properly loaded with the antibacterial therapeutic agent, rose bengal. The resulting microspheres were infiltrated into porous titanium substrate and sintered at 60 °C for 1 h, obtaining a convenient prophylactic network. In fact, the sintered polymeric microparticles were demonstrated to be key to controlling the drug dissolution rate and favoring the early healing process as consequence of a better wettability of the porous titanium substrate to promote calcium phosphate nucleation. Thus, this joint technology proposes a suitable prophylactic tool to prevent both early-stage infection and late-stage osseointegration problems.

## 1. Introduction

The use of materials with stiffness properties close to cortical bone has been widely investigated in the last decades to solve crucial problems, such as poor osseointegration and/or bacterial proliferation, that could occur at the implant/bone interface [1,2,3]. Although the search for new strategies to implement clinical implant performance continues to be a challenge for the scientific community, the use of porous titanium implants to resolve the stress shielding phenomenon arises as a widely accepted approximation. In this sense, the selection of the content, size, and degree of interconnectivity of the pores is critical not only to guarantee the biomechanical requirements but also to allow the growth of the bone toward the interior of the implant or effective infiltrations of coatings for therapeutic purposes [4].

It is known that osseointegration plays a key role in the earlier healing process and long-term durability to achieve clinical success [5]. Furthermore, other implant characteristics such as surface material properties, biomaterial composition, surface topography, mechanical overloading [6], as well as the presence of bacteria on the implant surface could hinder the osseointegration process. The early-stage issues occur right after the postoperative period and affect the healing; on the other hand, late-stage problems appear during the process of osseointegration. In particular, during the first few postoperative days, a surprising percentage of patients can present infections associated with edema, exudate, and pain [7]. Camps-Font et al. reported that up to 10% of the participants had early infections after dental implant placement [8] caused by microbial contamination during surgery through contact with the implants or from gloves or instruments [7]. Other authors reported [9] that the first 6 h after the surgery represent the target of an ideal antimicrobial activity release coating in order to protect the host while the immune system is weakened. Moreover, bacteria protected by biofilms, formed during the early-stage period, can require 1000 times the antibiotic concentration to be effective [9]. In addition, a study on antibiotic resistance in the human peri-implantitis microbiota demonstrated that 71.7% of the patients showed in vitro submucosal drug resistance to one or more of the antibiotics tested [10].

In order to bypass antibiotic resistance phenomena, some new chemicals have been designed against multidrug resistant (MDR) strains and appear as suitable alternatives in late-stage clinical studies [11]. For this purpose, although rose bengal (RB) has been legally approved just as a dye for the diagnosis of ocular disorders, it has recently been explored for its unique activity against cancer [12] and infections [13]. Recently, a work published in collaboration with Provectus Biopharmaceuticals reported that RB was very effective, and as an antimicrobial drug in skin and soft tissue infections (SSTIs), associated with Gram-positive bacteria and MDR strains [14]. Moreover, the therapeutic concentrations used in the study showed that RB has no systemic toxicological effects, mutagenic potential, or negative effects on female reproductive processes. RB is an anionic photosensitizer that produces singlet oxygen when exposed to light, which is considered its primary mode of anti-infective action under illumination [14]. The effect of RB can be modulated with photodynamic therapy (PDT) [13]. PDT has been used to treat periimplantitis to eliminate pathogens and promote crestal bone remodeling in patients affected by peri-implantitis associated with dental implantation [15,16]. The applicability of RB in dentistry was reported in a study, and it was demonstrated to reduce dentin collagen degradation [17].

Taking into account all these facts, several biodegradable dental implant coatings for controlled and targeted drug delivery have been evaluated as a therapeutic approach, since the elution time of the drug into the oral cavity is affected by the surface topographies of the devices and the amount of drug loaded [4]. In this sense, microspheres showed several advantages as a tailored strategy for biomedical purposes when compared to other formulations. In fact, they can be customized to obtain a larger surface area and uniform sizes/shapes to target the site/therapeutic effect, and to enhance drug loading ability to finally achieve a suitable drug release [18,19]. Microparticles based on biodegradable biopolymers have represented an optimal alternative as drug-delivery coatings for metallic dental implants. Full coating was shown to not be the best choice for implant-bone fixation, but microparticles have many advantages, as they favor bone healing due to their controlled particulate topography [20,21,22].

In this context, various polymers have been used to prepare microspheres for tissue engineering purposes, but aliphatic polyester poly(ε-caprolactone) (PCL) has been considered one of the best options, since its valuable features include stability in ambient conditions, cost, and the fact that it is readily available in large amounts [23]. Moreover, PCL has shown good mechanical properties, biocompatibility, non-toxicity, and slow biodegradability [24,25], and it has been approved by The Food and Drug Administration (FDA) for clinical use [26].

In this work, a novel solution is presented to jointly solve the problems of stiffness, osseointegration, and infections of titanium implants. Preparations of bioactive microspheres made up of polycaprolactone loaded with prophylactic therapeutic antibacterial, osseoinductive, and multidrug resistant RB, which could be infiltrated via a simple thermally sintered procedure on a porous titanium substrate manufactured using the space-holder technique, with a controlled pore content and size, were studied to guarantee the mechanical and clinical behavior of cortical bone tissue.

## 2. Materials and Methods

**Figure 1:**
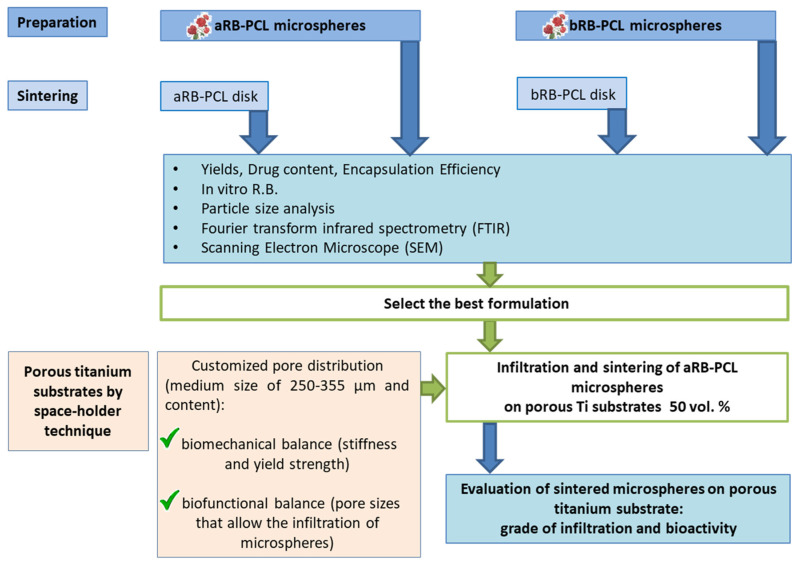
Workflow of the experimental work is shown in brief.

Figure 1 shows a schematic workflow of the experimental studies of this investigation. The preparation of the two formulations based on microspheres, named aRB-PCL and bRB-PCL, where RB was incorporated in a different way in the polymer, followed by their sintering in disks, represented the first part of the project. The comparison of their properties, obtained from characterization studies, led to the selection of the best microsphere formulation. The selected aRB-PCLs were infiltrated and thermally sintered in the porous titanium substrate, which was obtained by the space-holder technique with a medium pore size of 250–355 μm, and considered suitable for its biomechanical and biofunctional balance, as demonstrated by our previous studies [27]. Eventually, the infiltrated and sintered porous substrate was further explored for the evaluation of the required therapeutic effect.

### 2.1. Materials

Poly-ε-caprolactone (PCL, average Mn 80,000 g/mol) and polyvinyl alcohol (PVA, Mn = 30,000–70,000 g/mol, 87–90% hydrolyzed) were purchased from Sigma-Aldrich (Merck KGaA, Darmstadt, Germany). Rose bengal sodium salt (RB) and dichloromethane (CH_2_Cl_2_) were acquired from Sigma-Aldrich (St. Louis, MO, USA). Phosphate-buffered saline (PBS, NaCl 0.138 M; KCl 0.0027 M; pH 7.4; 25 °C) and hydrochloric acid 37% (HCl, EMSURE^®^ ACS, ISO, Reag. Ph. Eur. analytical reagent, Supelco^®^) were purchased from Sigma-Aldrich (Milan, Italy). Ultrapure bi-distilled water (H_2_O) was obtained by a MilliQ R4 system, Millipore (Milan, Italy). Commercially pure titanium (c.p. Ti) grade IV was supplied by SEJOING Materials Co. Ltd. (Seoul, Korea) with mean particle size d[50] = 23.3 µm [28]. Ammonium bicarbonate (NH_4_HCO_4_) (BA), 99% purity, supplied by Cymit Química S.L. (Barcelona, Spain), 250–355 µm in size, was used as spacer particles [29,30]. For the preparation of the solutions required by the titration method for the evaluation of bioactivity, sodium chloride (NaCl, BioXtra) ≥ 99.5% purity, di-sodium hydrogen phosphate dehydrate (Na_2_HPO_4_⋅2H_2_O, EMPROVE^®^ ESSENTIAL Ph Eur, BP, USP), calcium chloride (CaCl_2_) 97.0% purity, and Trizma base 99% purity (TRIS) were purchased by Sigma-Aldrich (Merck KGaA, Darmstadt, Germany).

### 2.2. Preparation of RB-PCL Microspheres

**Figure 2:**
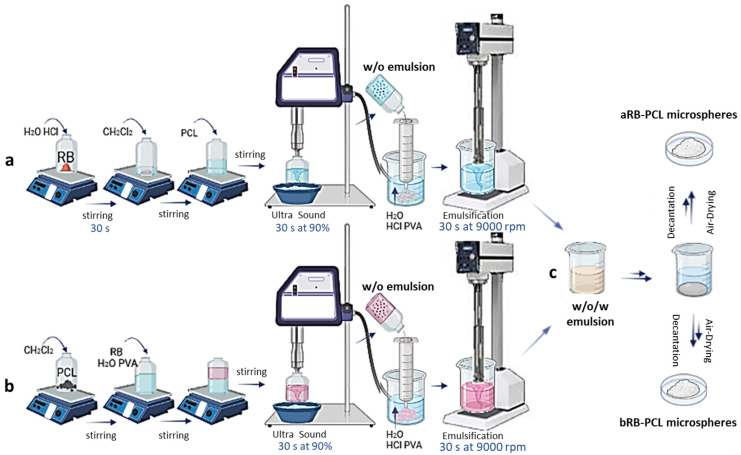
Schematic preparation of RB-PCL microspheres: (**a**) first part of aRB-PCL preparation; (**b**) first part of bRB-PCL preparation; (**c**) the last part of the process is in common, even carried out separately, resulting in aRB-PCL and bRB-PCL, respectively.

The RB-PCL microspheres were prepared using the double emulsion solvent evaporation technique (water/oil/water (w_1_/o/w_2_)) proposed by Luciani et al. [23], but appropriately modified to obtain two different microsystems (i.e., Figure 2: (a) aRB-PCL, and (b) bRB-PCL) based on H^+^ variations and RB solubility. In RB, salt is a water-soluble anion at a pH range of >5–12, but it is water-insoluble and organic solvent-soluble at acidic pH ranges [31].

The preparation of aRB-PCL was carried out as follows: A proper amount of RB red salt was added to a glass vial and dispersed in H_2_O with 0.1 M HCl. The dispersion obtained with a concentration of RB 5.4 mg/mL (w_1_) was gently stirred for 30 s and resulted in a dark red precipitate. PCL (60 mg/mL) in CH_2_Cl_2_ (o), with an w_1_/o ratio of 0.5/4.5 (*v*/*v*), was mixed with the phase w_1_. The vial was closed and magnetically stirred to obtain a white opalescent homogeneous system. Then, the obtained pre-emulsion was homogenized with a VC 50 (Sonics and Materials, Danbury, CT, USA) Vibra Cell probe sonicator for 30 s at 90% amplitude, and with the vial immersed in an ice bath. The resulting white primary emulsion (w_1_/o) was added dropwise, using a glass syringe (capacity 5 mL), to 125 mL of 0.5% (*w*/*v*) PVA solubilized in H_2_O with 0.1 M HCl, with a volume proportional to the ratio of w_1_/o/w_2_ of 0.5/4.5/125 (*v*/*v*/*v*). The secondary emulsion was achieved by using a Silverson SL2 mixer apparatus for 30 s at 9000 rpm (Silverson Machines, Inc., Waterside Chesham, UK) to produce a white double w_1_/o/w_2_ emulsion.

Microsphere hardening was achieved through the spontaneous evaporation of the CH_2_Cl_2_. The resulting solid white microparticles were easily separated by the decantation of the aqueous phase and air dried at room temperature in dark conditions. Finally, the solid particles were stored in a desiccator until the moment of their use. Figure 2a,c show a schematic representation of the process.

The preparation bRB-PCL significantly differed in the solubilization of the RB salt in the primary emulsion, since in this case the drug was directly solubilized in the neutral w_1_ media prior to addition to the o phase. RB salt dissolved in 0.5% (*w*/*v*) PVA in H_2_O at 5.4 mg/mL (w_1_) was added to the vial containing PCL (60 mg/mL) in CH_2_Cl_2_ (o), with a w_1_/o ratio of 0.5/4.5 (*v*/*v*), and gently stirred for 30 s to obtain a pink pre-emulsion. After being subjected to ultrasound, a pink primary emulsion (w_1_/o) was obtained. This primary emulsion was added dropwise to 125 mL of 0.5% (*w*/*v*) PVA/0.1 M HCl in H_2_O. The emulsification of the pink droplets was achieved using the mixer, and a white double w_1_/o/w_2_ emulsion was produced. The color change (from pink to white) was obtained thanks to homogenization with a Silverson mixer that exposed the pink drops to the acidic medium of w_2_. Solid white microspheres were obtained and stored, as previously reported for aRB-PCL. Figure 2b,c represent the preparation in brief. Table 1 summarizes the RB and PCL contents employed in the two formulations, the pH and the composition of w_1_ and w_2_ media, and the used w1/o/w2 ratios in order to highlight similarities and differences in the two preparations.

### 2.3. Preparation of Disks of RB-PCL Sintered Microspheres

In this case, 30 mg each of aRB-PCL and bRB-PCL were separately poured into two molds and heated in an oven (model 400, Memmert, Schwabach, Germany) at 60 °C for 1 h, following the procedures reported by Luciani et al. [23]. The obtained sintered disks were cooled down to room temperature and removed from the molds for further analysis.

### 2.4. Yield, Drug Content, and Encapsulation Efficiency of RB-PCL Microspheres

The production yields (YPx%, x = a or b) were calculated for each formulation as percentages according to described procedures by Rassu et al. [32]. Each dried RB-PCL formulation obtained at the end of the process was weighed. The resulting values in milligrams were divided by the total amount of material used to prepare the feed solution as follows (Equation (1)):

(1) $$\mathrm{YPx}\%=\frac{\mathrm{xRBPCLm}}{\mathrm{RB}+\mathrm{PCL}}\times 100$$

where xRBPCLm (x = a or b) are the actual amounts of microspheres obtained, and RB and PCL are the mg of drug and polymer employed in the process, respectively.

The real amount of drug loaded in the two different microspheres was assessed by calculating the drug content [32,33]. For this reason, microspheres were destroyed, as proposed by Tice et al. [34], and appropriately modified for RB-PCL particles. Fifty milligrams of each formulation were dissolved in 0.4 mL of DCM and then mixed with pH 7.4 phosphate buffer up to 5 mL, giving two separated phases. Then 0.1 mL of the pink aqueous phase was collected and analyzed by a UV spectrophotometer according to the method described in Section 2.5. Equation (2) was used for the calculation:

(2) $$\mathrm{DCx}\%=\frac{\mathrm{xRB}\;\mathrm{mg}}{\mathrm{xRBPCLm}\;\mathrm{mg}\;}\times 100$$

where DCx% is the drug content percentage, and xRB mg (x can be a or b) represents the actual amount of drug that was measured for each formulation; xRBPCLm mg (x = a or b) represents the 50 mg of each type of microsphere used for the evaluation.

The obtained DCs% were compared to the theoretical drug contents of 1% (i.e., 1 mg of RB for 100 mg of microspheres) for calculating the percentages of encapsulation efficiencies obtained for aRB-PCL and bRB-PCL microspheres (Equation (3)) [33].

(3) $$\mathrm{EEx}\%=\frac{\mathrm{DCx}\%}{\mathrm{DCt}\%\;}\times 100$$

where EE% is the percentage of encapsulation efficiency obtained for the systems (x = a or b) and DCt% is the theoretical drug content.

Values were determined by performing experiments in triplicate. The results obtained for each formulation are the average of three determinations (n = 3; ± standard deviation, SD).

### 2.5. Physical and Chemical Characterization of Microspheres

UV analysis was employed for the RB quantification. Evaluation of RB content in PBS buffer (pH 7.4), used for both the determination of the drug content (Section 2.4) and in vitro drug release studies (Section 2.5), was carried out with a UV spectrophotometer (Shimadzu UV-1800, Kyoto, Japan) (λ = 549 nm) [12]. RB concentrations were calculated by interpolation of the calibration curve prepared in PBS buffer (pH 7.4). Linearity (*y* = 0.0823*x* + 0.0095; *R*^2^ = 0.9994) was obtained with standard working solutions used in the range of 1–20 μg/mL.

The particle size of the microspheres was measured by the use of a Coulter laser diffraction analyzer, model LS 100Q (Beckman Coulter, Miami, FL, USA). HCl 0.1M was used as dispersion medium, and the sonication for the suspension was about 10 s. Measurements were made in triplicate on three samples of each formulation at room temperature under gentle magnetic stirring.

Fourier transform infrared spectrometry (FTIR) was used to understand the different incorporations of the drug in the two proposed formulations. The structural characterization of the samples (aRB-PCL, bRB-PCL, PCL, RB, and the physical mixtures of PCL with RB) was assessed. KBr disks were prepared as follows: 0.9% of bulk PCL in KBr, 0.1% of bulk RB in KBr, physical mixtures of 0.9% PCL with 0.1% RB, 1% of aRB-PCL microspheres, and 1% of aRB-PCL microspheres. FTIR spectra were acquired with a Nicolet Avatar 320 FTIR spectrometer (Nicolet Instrument Corporation, Madison, WI, USA) and recorded within the interval of 4000 to 400 cm^−1^.

Thirty milligrams each of aRB-PCL and bRB-PCL, and the sintered disks were immersed in 10 mL of PBS buffer (pH 7.4) at 37 °C, as previously reported by Bottino et al. [35], for the estimation of in vitro drug release from a dental implant surface modifier; sink conditions were ensured, and the aqueous solubility of RB is 100 mg/mL, according to *The Sigma-Aldrich Handbook of Stains, Dyes, and Indicators* [36]. Therefore, 1 mL aliquots were collected at different time points and subsequently transferred to a cuvette for further UV analysis; 1 mL of fresh PBS at 37 °C was replaced, allowing for the volume recovery. The concentrations of RB released from formulations in the PBS buffer (pH 7.4) were evaluated by interloping the obtained absorbance values obtained in the calibration curve (Section 2.5). The results were reported as the cumulative amount of RB released per unit of time. Values for each formulation were the average of three determinations belonging to three different samples (n = 9; ±standard deviation, SD).

### 2.6. Fabrication and Basic Characterization of Porous Titanium Substrate

Commercially pure (c.p.) porous titanium substrates (disks of ~12 mm diameter and 5 mm height) were prepared by the space-holder technique (SH), as previously reported in [37,38,39,40,41]. Briefly, ammonium bicarbonate (NH_4_HCO_3_) was mixed with the c.p. Ti (50 vol%) and pressed at 800 MPa using an Instron 5505 machine. The spacer particles were removed in a furnace under low vacuum of ~10^−2^ mbar throughout two temperature cycles (e.g., 333 K and 380 K) for 12 h each treatment. Subsequently, the sintering was carried out in a molybdenum chamber furnace (Termolab, Agueda, Portugal) under high vacuum conditions (~10^−5^ mbar) at 1523 K for 2 h. Next, the surface of the porous titanium disks was prepared following a standard metallography procedure (grinding and mechanical–chemical polishing).

The porosity of the titanium substrate was determined using the Archimedes’ method [42], from which the percentages of total and interconnected porosity (P_T_ and P_i_, respectively) were determined. Furthermore, image analysis (IA) was carried out on optical images acquired with a Nikon Eclipse MA100 N microscope to characterize the equivalent diameter of the pores, D_eq_, and the shape factor, F_f_ = 4πA/(PE)^2^, where A is the area of the pores, and PE is the experimental perimeter of pseudo-elliptic pores. For image analysis, Image-ProPlus 6.2 software (Mediacibernectic, Bethesda, MD, USA) was used. In addition, scanning electron microscopy images were also acquired with a FEI Teneo microscope (FEI, Eindhoven, The Netherlands) operating at 15 keV for structural characterization. This microscope was equipped with an energy-dispersive X-ray spectroscopy system (EDS-SEM) for compositional analyses. Finally, the mechanical properties of the porous c.p. Ti substrates, such as the yield strength and Young’s modulus, were estimated from the porosity data using equations already reported in the literature [37].

### 2.7. Coating and Infiltration of the Microspheres in the Porous Titanium Substrate

aRB-PCL was selected for all studies on a porous titanium substrate because the optimal profile was obtained during the in vitro drug release studies and scanning electron microscope (SEM) micrographs of the sintered microparticles. For this purpose, 30 mg of aRB-PCL particles were suspended in 0.5 mL of HCl 0.1M, sonicated for 10 s, and poured onto porous titanium disks using a heat shrink tube to ensure that all the solution penetrated in the substrate. Afterwards, the same conditions reported in Section 2.3 were adopted to obtain the required sintered samples for the wettability test. On the other hand, to evaluate in cross-section the adhesion of RB-PCL microspheres sintered on the porous titanium substrate by SEM, a D-shape of the porous titanium disk was infiltrated with a proper amount of aRB-PCL and exposed to the same treatment mentioned above. The D-shape was examined by SEM both from the top to determine the homogeneous distribution of the sintered microparticles, and from a cross-section view to check how the microspheres infiltrated the porous substrate (Section 3.3).

### 2.8. Morphological Analysis by SEM

The morphology of the microspheres and the sintered matrices (before drug release studies and after RB release) was investigated by SEM using a Phenom Pro Desktop Scanning Electron Microscope (Phenom, Eindhoven, The Netherlands). This instrument allowed the direct observation of samples without pretreatment using a temperature-controlled support that froze microspheres and sintered matrices at 20 degrees below zero and in low vacuum conditions.

### 2.9. Osteoactivity Evaluation of Sintered Microspheres by the Use of Wettability

The wettability profile of the sintered microspheres on the porous titanium substrate, obtained as reported in Section 2.7, was determined. The porous substrate was analyzed both without coating and with sintered aRB-PCL microspheres. The contact angle of an HCl 0.1M water droplet was estimated using an SEO Phoenix Contact Angle Analyzer (Kromtek Sdn Bhd, Selangor, Malaysia) for the evaluation of the early osseointegration activity [43].

### 2.10. Calcium Phosphate Nucleation In Vitro Test

The evaluation of the potential bioactivity of the sintered microspheres in vivo was carried out using a rapid in vitro protocol based on the determination of the calcium phosphate nucleation onset point related to the evolution of pH by following Zhao et al. [44]. A calcium solution was added (0.1 mL/min) using a 1 mL syringe to 50 mL of phosphate solution, maintained under stirring, containing a disk of aRB-PCL microspheres, or without testing material for the blank-control comparison, until calcium phosphate nucleation took place, as monitored by a pH electrode (pH 50+DHS Set bench meter incl. pH-electrode 201T, Dostmann Electronic GmbH, Germany). The employed solutions were prepared as Zhao reported [44]. Calcium stock solutions contained 100 mmol NaCl, 20 mmol CaCl_2_, 20 mmol Tris, and 17.5 mL of 1.0 M HCl solution per liter of solution, and phosphate solutions were made up of 100 mmol NaCl, 4 mmol Na_2_HPO_4_·2H_2_O, 20 mmol Tris, and 17.5 mL of 1.0 M HCl solution per liter of solution. During the experiment, which lasted 270 min, the temperature was controlled at 25.0 ± 1.0 °C. The initial pH of the solution was experimentally measured and was 7.45 and was monitored in situ by the pH electrode. A disk of 100 mg of aRB-PCL, obtained as reported in Section 2.3, was exposed to the treatment.

### 2.11. Statistical Analysis

The yielded data, encapsulation efficiency results, and particle size profile of the two formulations were analyzed using the nonparametric Mann–Whitney test. Drug content data were evaluated and compared with the theoretical value (1%) using the nonparametric Kruskal–Wallis test, and individual differences were analyzed using a post hoc Dunn’s multiple comparison test. Evaluation of the in vitro drug release data was performed using the two different nonparametric tests. The Mann–Whitney test was used for the evaluation of the 100% (released RB) time point. On the other hand, the Friedman test was used to compare the different formulations, and individual differences were analyzed using a post hoc Dunn’s multiple comparison test. Wettability data were analyzed using ordinary one-way ANOVA. The calcium phosphate nucleation in vitro test was analyzed using the nonparametric Mann–Whitney test. GraphPad Prism (version 8.01; GraphPad Software Incorporated, San Diego, CA, USA) was used for all analysis. Statistical significance was established at *p* < 0.05.

## 3. Results and Discussion

### 3.1. Manufacturing and Characterization of Microspheres and Sintered Disks

#### 3.1.1. Comparison of the Two Processes

The yields of production, drug content, and encapsulation efficiencies of aRB-PCL and bRB-PCL gave optimum results. These data confirmed that the double emulsion solvent evaporation technique employed was a suitable method for the preparation of both formulations, where RB was solubilized or suspended in the water of the primary emulsion depending on pH changes. Despite the low solubility of the RB salt in organic solvents (logP, log partition coefficient octanol/PBS = 0.59 [13]) and the low affinity for the lipophilic PCL, the method resulted in very good results in terms of the amount of drug loaded into the microspheres.

**Figure 3:**
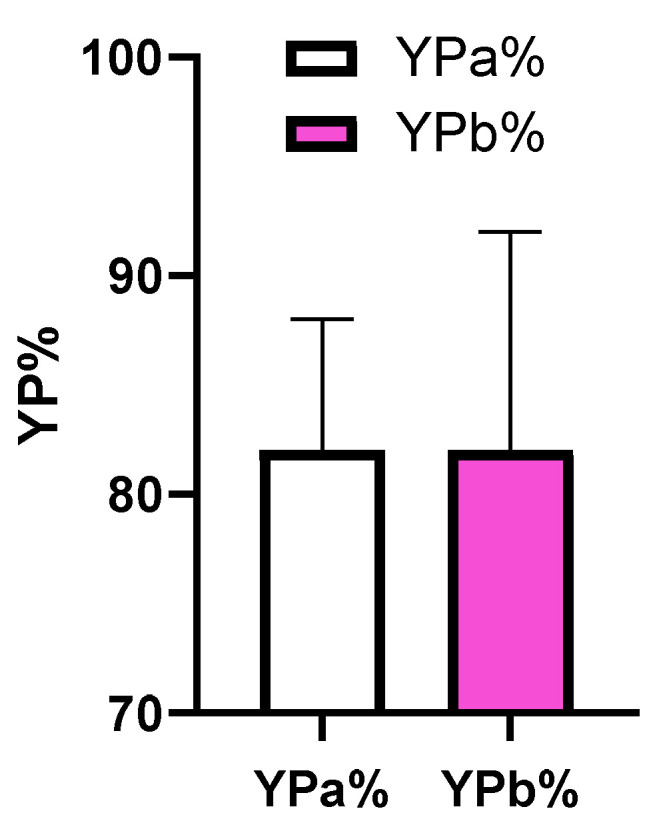
Yields of production for aRB-PCL (YPa%) and bRB-PCL (YPb%).

**Figure 4:**
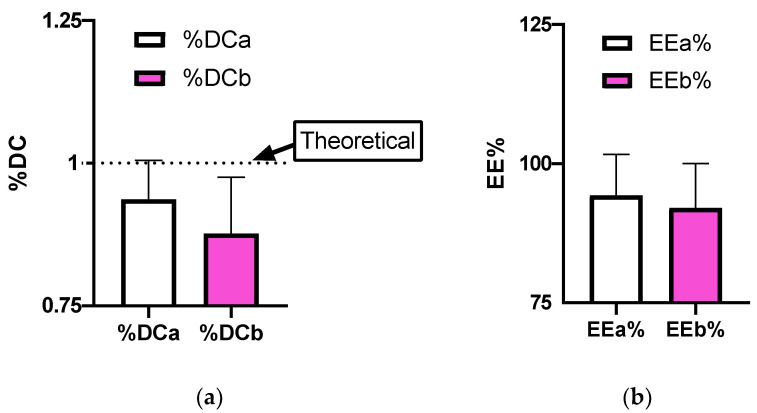
Representation of obtained results for aRB-PCL and bRB-PCL about (**a**) drug content (%DC); and (**b**) encapsulation efficiency (EE%).

Microparticles resulted in production yields exceeding the mean of 80% for both formulations (Figure 3); YP% values were not significantly different (*p* = 0.6000). Real drug contents (Figure 4a) were very close to theoretical values of 1% (*p* > 0.05). Moreover, encapsulation efficiency values exceeded the 90% for both formulations (Figure 4b) without statistical differences (*p* = 0.8000).

#### 3.1.2. Size and Morphology

**Figure 5:**
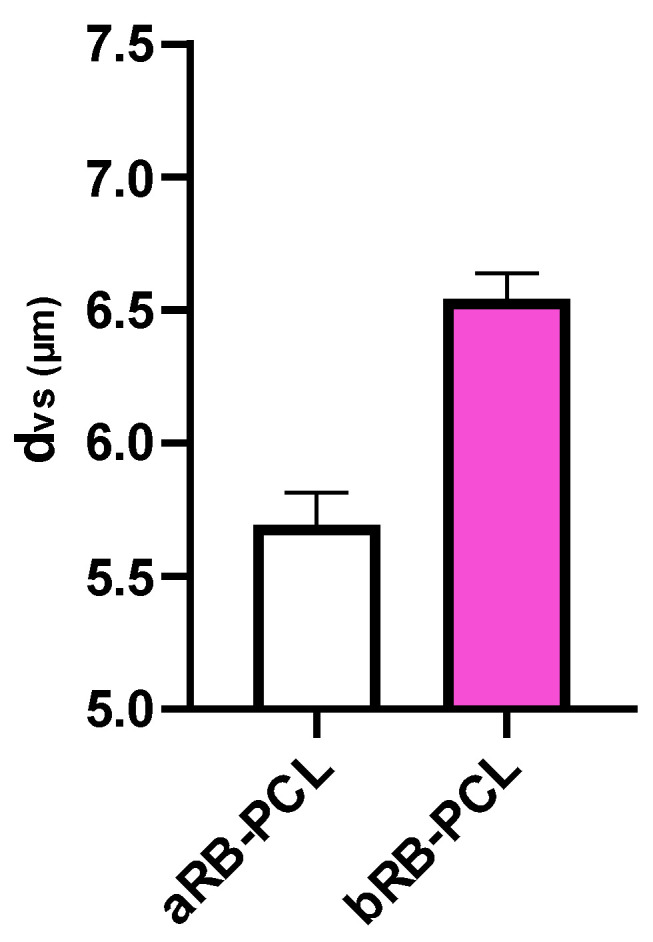
Graphical representation of volume-surface diameters (d_vs_) of aRB-PCL and bRB-PCL.

The volume-surface diameter (d_vs_) results for the two formulations are reported in Figure 5. No significant differences were observed between the two groups (*p* > 0.05). The distribution curves of the aRB-PCL and bRB-PCL showed a very similar profile and were characterized by small d_vs_ values of 5.69 ± 0.12 and 6.54 ± 0.10 μm, respectively, and preponderant unimodal particle size distributions considering the SD values (Supplementary Figure S1), even if the presence of a smaller sized population could be observed. The method allowed for the obtaining of customized microparticles with the proper sizes for their subsequent infiltration in the porous substrates due to pores of 250–355 μm.

**Figure 6:**
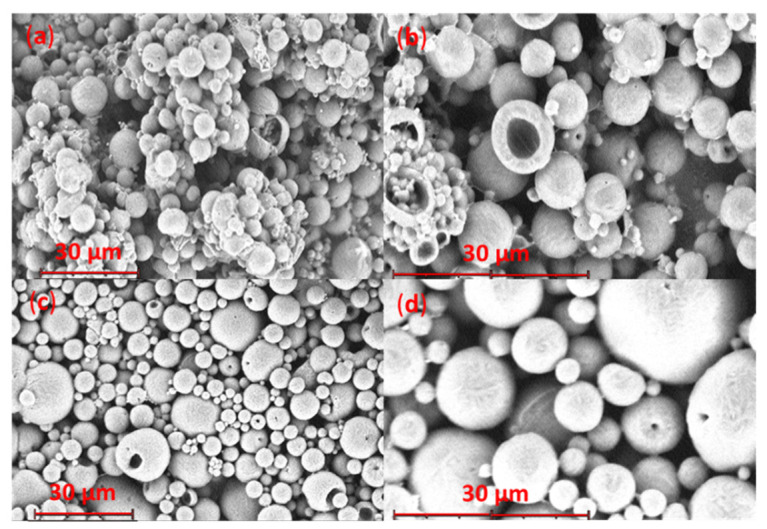
SEM micrographs of RB-PCL microspheres, obtained at different magnifications: (**a**) aRB-PCL at 2350×; (**b**) aRB-PCL at 4700×; (**c**) bRB-PCL 2350×; (**d**) bRB-PCL 6700×.

The aRB-PCL was quite homogenous, spherical, and not porous, with a surface that appeared slightly rough, as shown in Figure 6a,b. Some of them had a few very small holes due to the vacuum applied during the analysis. The smaller size population resulting from Coulter analysis was present in a very small amount also in the micrographs. These microparticles appeared quite similar to those obtained by Luciani et al. [23], loaded with a protein distributed inside the matrix, even if they had bigger sizes.

No significant morphological distribution changes were observed in the case of the bRB-PCL (Figure 6c,d). SEM images showed spherical and not porous microparticles with a rough surface but were slightly different from those where RB was solubilized in CH_2_Cl_2_ within the polymer, supporting the different incorporation of RB in the two formulations.

**Figure 7:**
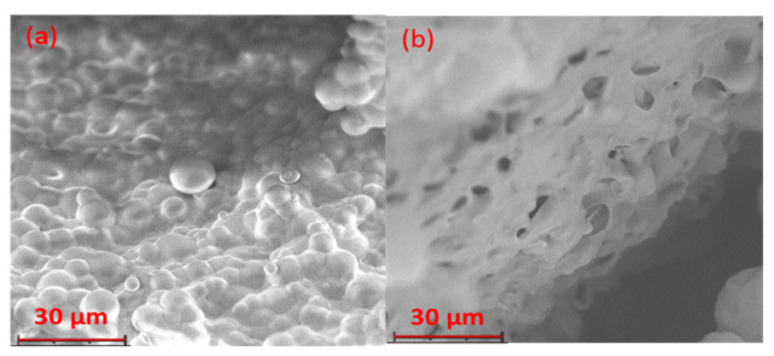
SEM micrographs of microparticle exposed to sintering obtained at 2350× magnification: (**a**) aRB-PCL sintered disk; (**b**) bRB-PCL sintered disk.

The two families of microparticles analyzed after the sintering, which gave microsphere disks, had distinct morphologies (Figure 7). The aRB-PCL disk maintained an identifiable and spherical shape of microparticles in the sintered matrix. On the other hand, the bRB-PCL disk showed multiple invaginations in the homogenous sintered matrix, with no identifiable microparticles.

#### 3.1.3. FTIR Spectroscopy

FT-IR spectra of the crystalline drug, polymer, and the physical mixtures of PCL with RB, aRB-PCL, and bRB-PCL were recorded (Supplementary Figure S2). Both microparticles and the physical mixtures presented typical PCL peaks at 2938, 2861, 1735, 1288, 1235, and 1185–1165 cm^−1^ [45]. However, there was a clear difference in the profile for all of these peaks in the aRB-PCL when compared to those of the physical mixtures and the other formulation. In addition, similar behavior was observed for the distinguishable peak of RB at 3427 cm^−1^ (-OH) [46]. These results highlighted the different incorporation of RB in the two PCL-based microparticles, and the lower intensity of the signals in the aRB-PCL indicated that RB was well incorporated into the matrix [47].

#### 3.1.4. In Vitro Drug Release Studies

**Figure 8:**
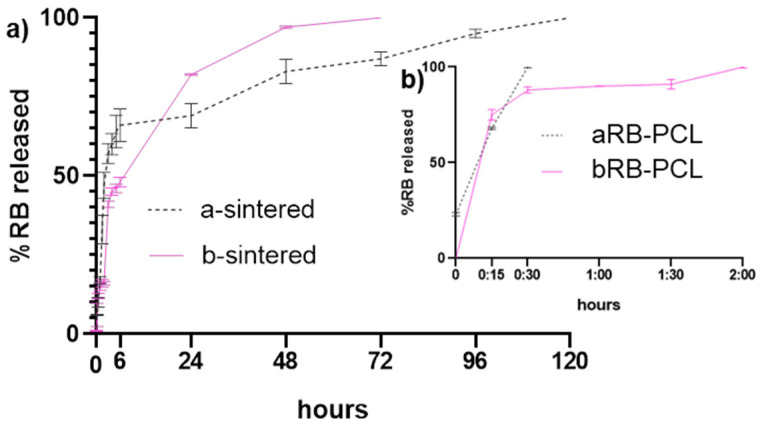
Graphical representation of in vitro release study on: (**a**) aRB-PCL and bRB-PCL; (**b**) aRB-PCL disks (a-sintered) and aRB-PCL disks (b-sintered).

Table S1 shows the results of the in vitro drug release tests of the microspheres in phosphate buffer (pH 7.4) at 37 °C compared to the dissolution behavior of their sintered materials. Both formulations, based on microspheres and sintered systems, controlled rose bengal release during a period of time as a consequence of the pH stimuli in physiological conditions. In fact, it was noticed by the UV analysis of the decanted solution that 0% of RB was released from the microspheres under acid conditions (0.1 M HCl) of the water medium of the second emulsion (w_2_), where finally the microspheres, prior to their drying, were obtained. However, while approximately 100% of the drug was released from aRB-PCL and bRB-PCL (Figure 8a) within 0.5 h (30 min vs. 1 h, *p* > 0.05) and 2 h (2 h vs. 3 h, *p* > 0.05), sintered formulations showed a prolonged release within a few days (Figure 8b). In particular, the two sintered microspheres presented the expected [48] and required [9] burst release during the first 6 h (i.e., 66% for a and 48% for b), but the a-sintered formulation was followed by a slow and continuous release up to 5 days, while the b-sintered formulation behaved with a “step-profile” and ended the release in 3 days. Data analysis was carried out by comparing the release profile of RB-PCL microspheres with their own sintered materials; results provided a significant difference in both cases (*p* < 0.05), but no significant differences were found in which the dissolution behavior of the two types of microspheres matched each other (*p* > 0.05). The profiles obtained for the sintered microspheres were in good agreement with the behavior required for a drug delivery system for dental implants [48]. However, the long-lasting release of RB from aRB-PCL sintered microspheres for antibacterial activity [14] was the parameter of selection for further analysis in the porous titanium substrate.

Since the extensive study by Kurosu and colleagues has been recently reported [14] on the antibacterial activity of RB, in this work we decided to correlate the results of our in vitro release study with their tabulated MICs to demonstrate the potential applicability of the selected formulation in dental implant infections. In fact, Kurosu demonstrated that RB was effective against Gram-positive bacteria, even drug-resistant microbiota, and able to eradicate biofilm at low concentrations (0.01–3.13 μg/mL) under fluorescent, LED, and natural light in a few minutes. In particular, we evaluated the actual possibility of using RB under LED (in PDT therapy) or dark conditions in response to the dosage released between the subsequent checked aliquots. Figure S3a reports the concentrations, expressed in ppm, of RB released and demonstrated that under LED conditions, 30 mg of our sintered aRB-PCL is effective to kill, starting from around 0 min, *Staphylococcus aureus* BR 5 (A methicillin and vancomycin resistant strain), *Staphylococcus aureus* strain AIS 1,000,505 (VRS10, a vancomycin-resistant strain), *Staphylococcus aureus* USA100 strains 71,080 (VRS8, a vancomycin resistant strain), *Streptococcus salivarius* subsp. *salivarius* 7073™, *Staphylococcus epidermidis* 35984^TM^, among many others, since these bacteria showed a MIC between 0.39 and 0.78 ppm. In dark conditions, 300 mg of sintered material was required to obtain the same great activity after 15 min. Moreover, the highest concentrations were achieved during the first 6 h (Figure S3b) in good accordance with the behavior required for implant coating drug release systems [9].

**Figure 9:**
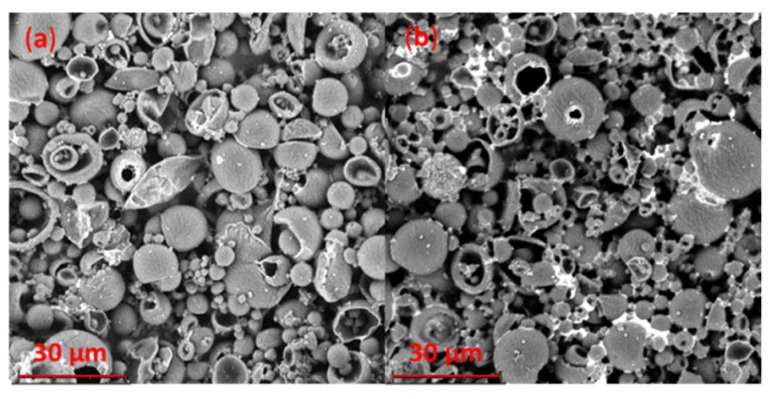
SEM micrographs of microparticles exposed to the in vitro drug release studies, obtained at 2350× magnification: (**a**) aRB-PCL; (**b**) bRB-PCL.

A similar behavior was noticed after the analysis of the microparticles exposed to in vitro release studies. Both formulations showed important microstructural and morphological changes as a result of the rapid dissolution of the drug entrapped in the matrix. The formation of big holes within irregular surfaces and non-spherical appearance took place (Figure 9).

**Figure 10:**
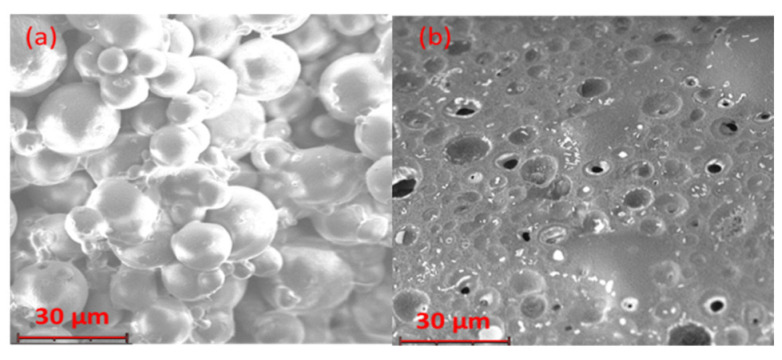
SEM micrographs of sintered disks analyzed after the in vitro drug release studies, obtained at 2350× magnification: (**a**) aRB-PCL sintered disk; (**b**) bRB-PCL sintered disk.

RB-PCL disks after in vitro drug release studies are shown in Figure 10. Micrographs showed the absence of large external holes in the aRB-PCL sintered disk also after the release of the drug. Nevertheless, remarkable features in the microstructural morphology could be observed. Variations in the sintered-microparticle interconnections, due to the release of the drug, resulted from the comparison, at 2350 times magnification, of the micrographs in Figure 9a with Figure 10a. On the other hand, a surface rich in cavities was presented by the bRB-PCL sintered disk in Figure 10b, with the same test, due to the dissolution of the RB, in good agreement with the irregular surface shown in Figure 9b. This different appearance for the two formulations, obtained after the sintering, confirmed the important effect of the sintering process on the microspheres that influenced the drug release. Moreover, the divergent entrapment of the drug in the two formulations, previously demonstrated by FT-IR analysis, was also supported by SEM micrographs.

Considering both the best results obtained from the drug release studies, where 100% of the drug was found in solution at 5 days (versus 2 days of formulation b), and the morphological aspects shown by SEM micrographs of the sintered disk, the formulation selected as the best system for further infiltration in the porous titanium substrate was aRB-PCL.

### 3.2. Characterization of the Porous c.p. Ti Substrates

Scanning electron microscopy and laser confocal microscopy images of the porous titanium substrate before being coated with the microspheres are presented in Figure S4.

Small micropores (around 10 μm) inherent to the sintering process and the macroporosity associated with spacer particles (>200 μm) were observed. The macro-pores allowed the infiltration of the polymeric microspheres. The S_a_ (30.8 ± 0.2 μm) parameter, measured on laser confocal microscopy images, revealed a high roughness for the porous titanium substrate, as expected. The SEM images of the inside of the macropores and the rough wall surface of the pores could be appreciated. The total and interconnected porosity substrate (P_t_ = 48.5 ± 0.9 and P_i_ = 42.8 ± 0.7) were obtained by the Archimedes’ method and were close to 50 vol%, while the pore size (D_eq_ = 260 ± 22 μm) measured by image analysis (IA) had a value between the range of the spacer particle size. These results corroborate the effectiveness of the space holder technique as a fabrication process to tailor the desired porosity. The porous Ti disk used had an appropriate balance between porosity, which favors osseointegration, and the resulting biomechanical properties, as described in detail in [40,41,43,49,50]. The type of substrate used in the present manuscript shows an adequate biomechanical and biofunctional balance to replace small areas of cortical bone, matching the requirements of these tissues (E = 20–25 GPa y S_y_ = 150–180 MPa).

#### Wettability of Porous Titanium Substrates

The contact angle of the substrate prior to infiltration and sintering of the microspheres was 48.74 ± 2.14°, demonstrating a quite hydrophilic behavior. A detailed analysis is reported in Section 3.3 comparing this result to those obtained after infiltration of the substrate and the tabulated value for the bulk PCL.

### 3.3. Infiltration of Porous Titanium Substrate and Characterization

The infiltration and sintering of the porous titanium substrate with the selected microparticles allowed for the evaluation of how the microsystem penetrated its holes, as well as their distribution on the surface, which also modified the hydrophilic behavior of the metallic disk.

**Figure 11:**
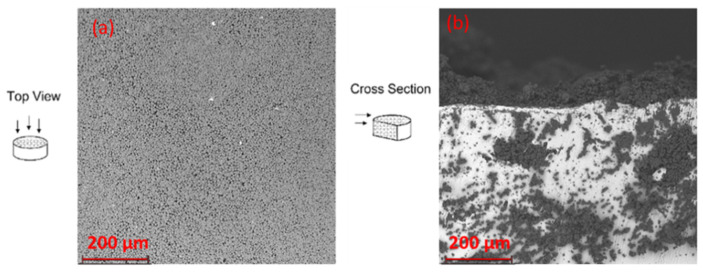
SEM micrographs of aRB-PCL infiltrated in porous titanium substrate and exposed to sintering: (**a**) top view obtained at 350× magnification; (**b**) cross-section view obtained at 390× magnification.

SEM micrographs (Figure 11) show the D-shaped treatment with the PCL microspheres loaded with RB infiltrated in the substrate and eventually thermally sintered. The top view illustrates that the microparticles maintained the spherical shape and adhered perfectly and homogenously to the substrate. The cross section allows for viewing how pores of the white titanium substrate were completely and uniformly filled by the sintered microparticles. It can be noted that microparticles allowed for deep infiltration of the porous substrate. This was due to the presence of inner channels related to controlled porosity, which formed connections between the pores. These results also confirmed the optimum ability of the selected porous titanium substrate to allow the infiltration of biomaterials, as previously reported by these authors [27].

**Figure 12:**
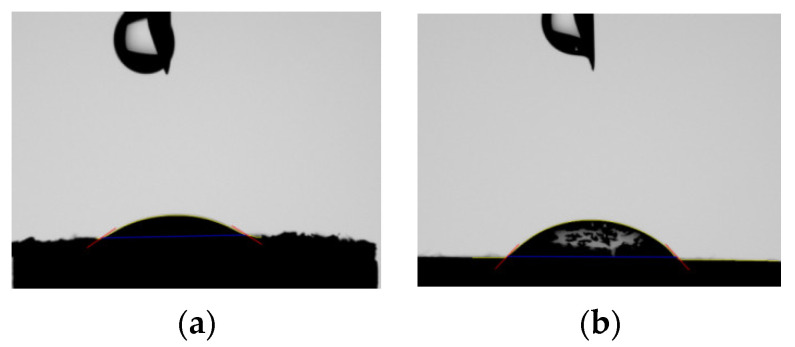
Contact angles of (**a**) aRB-PCL infiltrated and sintered in porous titanium substrate and (**b**) in porous titanium substrate.

The microspheres loaded with RB, infiltrated, and sintered on the porous titanium disk presented a more hydrophilic behavior (mean contact angle 32.98 ± 0.61°) than only porous titanium substrate mean contact angle (48.74 ± 2.14°). These angles were compared to the contact angle of bulk PCL (101 ± 3°) [51] using one-way ANOVA for the evaluation of the different wettability of PCL-microspheres, the very hydrophobic bulk material, and the porous titanium substrates; *p* < 0.0001 demonstrated a significantly different behavior of our materials and PCL. The reduction in the contact angle observed when coating with microspheres could respond to the wetting effect provided by the PVA added as a stabilizer and the microtopography created by the sintered microspheres. The contact angles between the drop solution and our two different surfaces are shown in Figure 12. Results confirmed the appropriate use of microparticles instead of a continuous layer of bulk PCL to perform the bioactivity of this porous titanium substrate that has previously been shown to prompt bone healing [38,40,52].

It is well known that hydroxyapatite (HA) is the stable biological mineral form of calcium apatite that triggers osteoblast activity for bone generation [4,53,54]. Therefore, the investigation of the potential of a material to induce HA growth onto its surface is a determining factor to estimate the capacity this material presents to promote osseointegration. Most studies about HA growth are based on long experiments following ISO 23314:2014, which requires maintaining the samples immersed in SBF (simulated body fluid) for 28 days. However, the method presented by Zhao et al. [44] is an interesting alternative to assess in a few hours the potential of the material to generate HA by correlating the evolution of the pH of a phosphate solution containing the testing material to determine the calcium phosphate nucleation onset point. They demonstrated that each point in the pH profile, highlighted by a distinct visible variation of slope in the pH evolution, corresponds to a sudden decrease of available free Ca^2+^. In particular, the first point corresponds to the formation of octacalcium phosphate (OCP), and the second one is the result of HA formation; this is the more stable form that reflects the bioactivity behavior of the material.

The results obtained in the in vitro test for the qualitative evaluation of the ability of the a-sintered disk to initiate the HA nucleation are graphically reported in Figure S4. From the blank control curve (Figure S5) obtained in our work, the very same clear two-step profile observed by Zhao et al. can be noticed. In addition, OCP and HA formation took place at around 125 min and around 150 min, respectively. Moreover, Zhao demonstrated that the faster these two points are achieved, the better the nucleation activity was shown. In this work we statistically compared the curve profile obtained in the presence of a sintered sample to the curve obtained in the absence of a sample (blank). The nonparametric Mann–Whitney test gave *p* < 0.0001, demonstrating the significant difference in the pH profile, corresponding to a sudden decrease in pH, obtained when the test was performed in the presence of 100 mg of the a-sintered disk.

## 4. Conclusions

The results presented in this work show a simple, cheap, suitable, and bioactive alternative to overcome common implant/bone interface issues such as bacterial proliferation, the stress shielding phenomenon, and poor osseointegration. RB-loaded microspheres based on biocompatible and non-toxic PCL can be easily obtained by the double-emulsion solvent evaporation technique. The loading of the drug into the microparticulate and sintered network resulted in a significant prolonged in vitro release, providing an antimicrobial effect against MDR strains in dark conditions, or PDT therapy, for 5 days. Good results in terms of wettability and HA nucleation, as well as SEM micrographs demonstrated that the infiltration of the formulation, followed by thermal sintering in the porous substrate, allows for the possibility of using this coating on titanium samples fabricated ad hoc, whose good biomechanical properties and biofunctionality are widely confirmed.

## Figures and Tables

**Table 1:** Formulative parameters employed for the preparation of RB–PCL microspheres.

	RB Content in w_1_ (mg/mL)	w_1_ Media	pH w_1_ Media	PCL Content in o (CH_2_Cl_2_) (mg/mL)	w_2_ Media	w_1_/o/w_2_ Ratio (*v*/*v*/*v*)
**aRB-PCL**	5.4	0.1 M HCl in H_2_O	1	60	0.1 M HCl 0.5% PVA in H_2_O	0.5/4.5/125
**bRB-PCL**	5.4	0.5% PVA in H_2_O	6.9	60	0.1 M HCl 0.5% PVA in H_2_O	0.5/4.5/125

## Data Availability

Not applicable.

## Supplementary Materials

The following supporting information can be downloaded at: https://www.mdpi.com/article/10.3390/pharmaceutics14061244/s1. Figure S1: Distribution curves: (a) aRB-PCL; (b) bRB-PCL. The equivalent spherical diameter was of 18.2 ± 0.5 µm and 19.9 ± 0.7µm, respectively. Figure S2: FTIR spectra of (a) RB 0.1%, (b) PCL 0.9%, (c) physical mixtures of PCL 0.9% with RB 0.1%, (d) aRB-PCL 1% and (e) bRB-PCL 1% are reported within the groups stretching. Structural formula for (f) the drug and (g) the polymer is showed in red and black, respectively. Intensity of signals is measured as the percent transmittance (%T). Figure S3: Graphical representation of Rose Bengal concentrations (ppm) released between subsequent studied time-points: (a) time-span of 120 h; (b) time-span of 6 h. Figure S4: SEM and confocal laser images of fully-dense, FD and porous substate (50 vol.% and 250–355 µm). Figure S5: Shows the curves on the pH evolution for the blank (black line) and the sample (pink line): It can be noticed that the presence of the disk does not appear to influence the calcium phosphate formation pathway because the curves have the same shape. The two-step process (i.e., OCP and HA formation) is delimited by both the light blue line and the dark blue line, respectively. Table S1: Percentages of RB (%) released during the time from in vitro drug release studies for the formulations in phosphate buffer (pH 7.4) at 37°C (mean ± SD).
