# Supplementary material for: Novel Utilization of Therapeutic Coatings Based on Infiltrated Encapsulated Rose Bengal Microspheres in Porous Titanium for Implant Applications
Source: Pharmaceutics. 2022 Jun 12;14(6):1244. doi: 10.3390/pharmaceutics14061244 (PMC9230760; doi:10.3390/pharmaceutics14061244)
Supplement: Supplementary file 1 [file pharmaceutics-14-01244-s001.zip › pharmaceutics-1758737-supplementary.pdf]

Novel utilization of therapeutic coatings based on infiltrated encapsulated Rose Bengal microspheres in porous titanium for Implant Applications

Francesca Accioni[1,2], Giovanna Rassu*[2], Belén Begines [1], Luisa Marleny Rodríguez Albelo[3], Yadir Torres Hernández[3], Ana Alcudia*[1] and Elisabetta Gavini[2]

[1] Departmento de Química Orgánica y Farmacéutica, Facultad de Farmacia, Universidad de Sevilla, Sevilla, España; francesca.accioni@gmail.com (FA); bbegines@us.es (BB); aalcudia@us.es (AA)
2 Department of Medical, Surgical and Experimental Sciences, University of Sassari, Sassari, Italy; eligav@uniss.it (EG); grassu@uniss.it (GR)
3 Departmento de Ingeniería y Ciencia de los Materiales y del Transporte, Escuela Politécnica Superior, Universidad de Sevilla, Sevilla, España; ytorres@us.es (YT); lralbelo@us.es (LMRA)
*Correspondence: aalcudia@us.es (AA), grassu@uniss.it (GR)

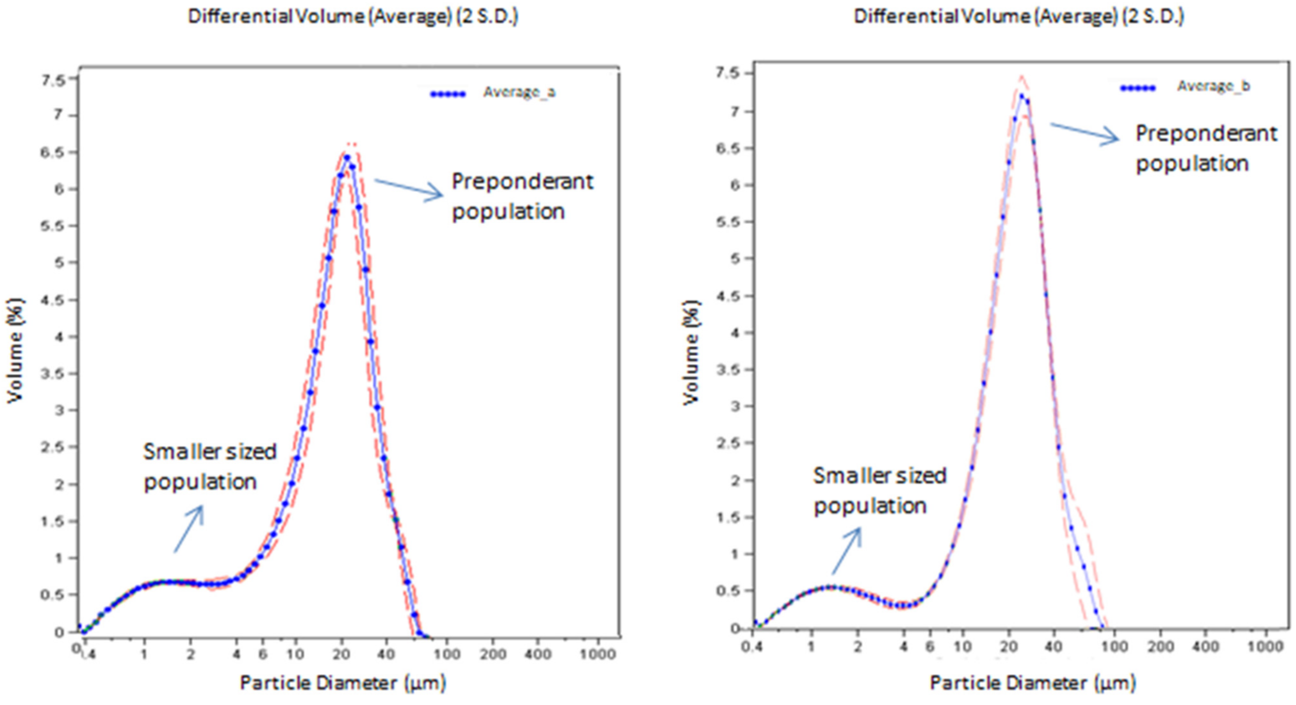

(**a**) (**b**)

Figure S1. Distribution curves: (a) aRB-PCL; (b) bRB-PCL. The equivalent spherical diameter was of 18.2 ± 0.5 µm and 19.9 ± 0.7µm, respectively.

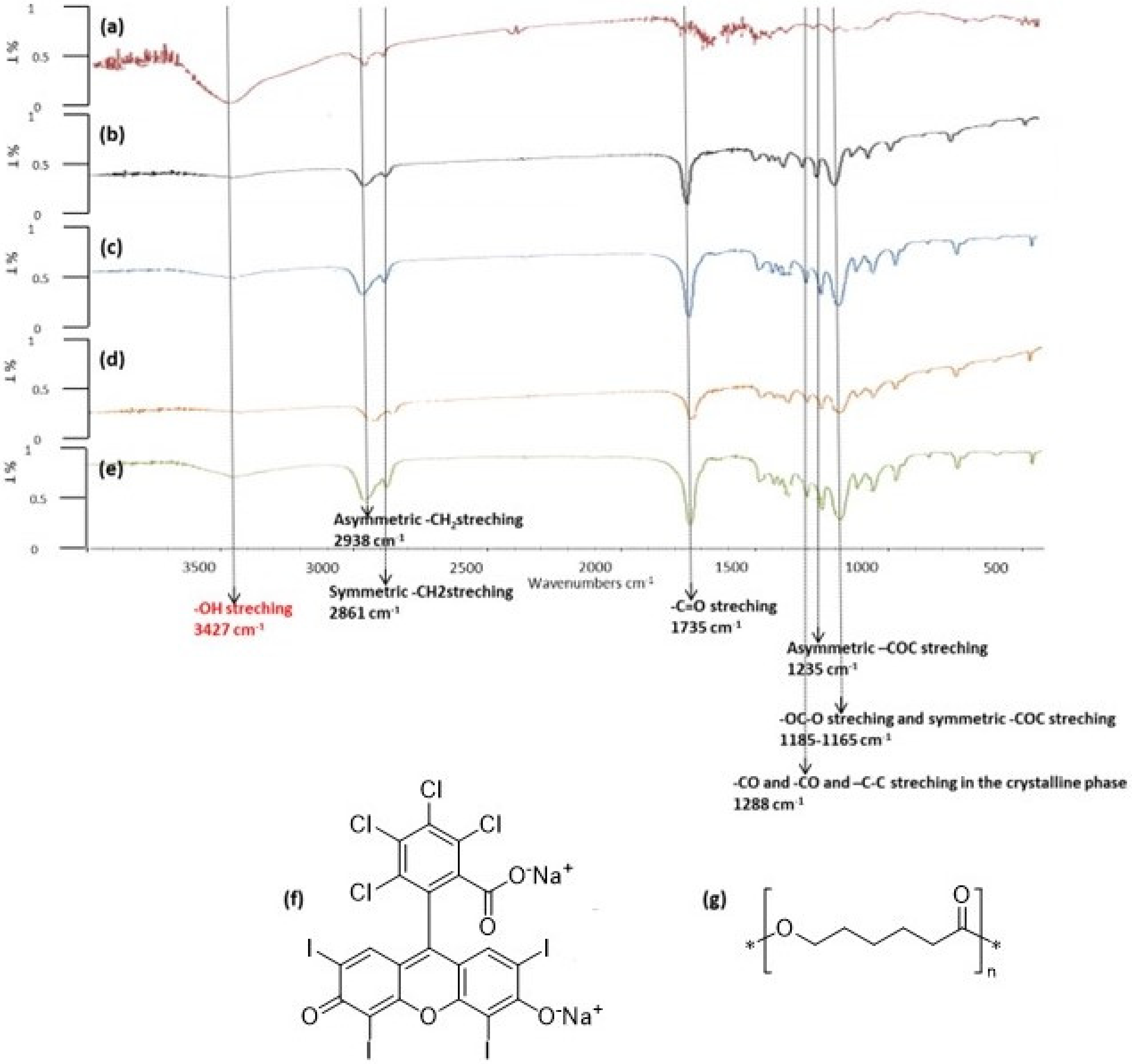

Figure S2. FTIR spectra of (a) RB 0.1%, (b) PCL 0.9%, (c) physical mixtures of PCL 0.9% with RB 0.1%, (d) aRB-PCL 1% and (e) bRB-PCL 1% are reported within the groups stretching. Structural formula for (f) the drug and (g) the polymer is showed in red and black, respectively. Intensity of signals is measured as the percent transmittance (%T).

Table S1. Percentages of RB (%) released during the time from *in vitro* drug release studies for the formulations in phosphate buffer (pH 7.4) at 37°C (mean ± SD).

Time (hours)	aRB-PCL (%RB)	bRB-PCL (%RB)	aRB-PCL sintered (%RB)	bRB-PCL sintered (%RB)
0:00:00	22.5 ± 0.9	0 ± 0	2.3 ± 0.1	1.5 ± 0.2
0:15:00	67.6 ± 0.6	75.1 ± 2.8	6.0 ± 0.2	6.4 ± 3.7
**0:30:00**	**99.6 ± 0.4**	87.6 ± 1.6	9.6 ± 1.5	12.5 ± 0.9

1:00:00	90.0 ± 0.2	17.4 ± 1.1	15.1 ± 1.3
1:30:00	91.3 ± 2.5	33.4 ± 4.5	15.7 ± 1.3
**2:00:00**	**99.5 ± 0.5**	46.8 ± 4.1	15.6 ± 0.6
3:00:00		56.9 ± 3.1	40.9 ± 1.0
4:00:00		60.0 ± 3.4	44.5 ± 1.2
5:00:00		63.7 ± 5.1	45.7 ± 1.4
**6:00:00**		**65.9 ± 5.2**	**47.6 ± 1.5**
24:00:00		69.4 ± 3.9	82.4 ± 0.3
48:00:00		82.6 ± 3.9	96.9 ± 0.4
**72:00:00**		87.0 ± 2.2	**100.0 ± 0.0**
96:00:00		94.8 ± 1.4	
**120:00:00**		**100.0 ± 0.0**	

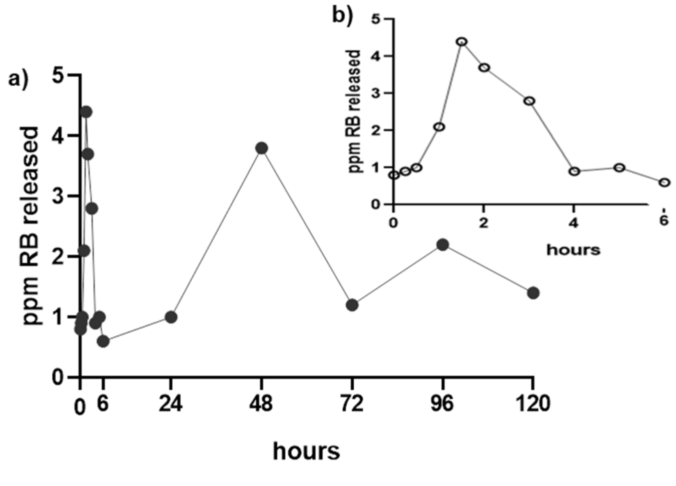

Figure S3. Graphical representation of Rose Bengal concentrations (ppm) released between subsequent studied time-points: a) time-span of 120 h; b) time-span of 6h.

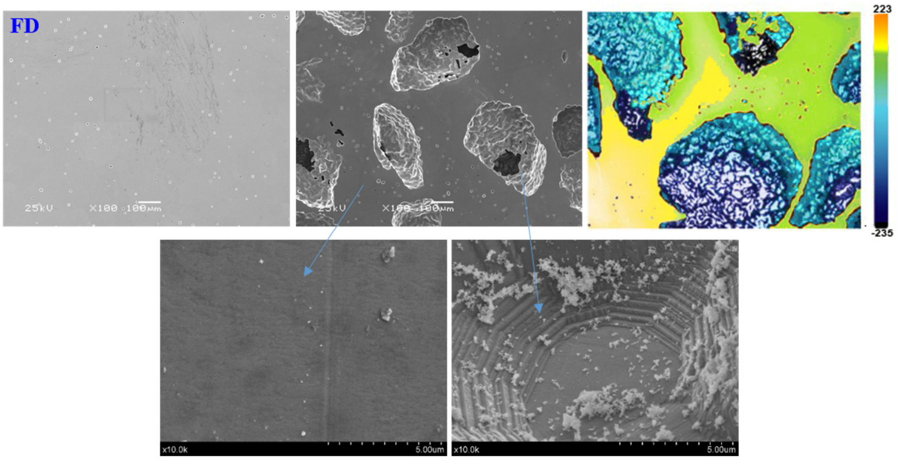

Figure S4. SEM and confocal laser images of fully-dense, FD and porous substate (50 vol.% and 250–355 μm).

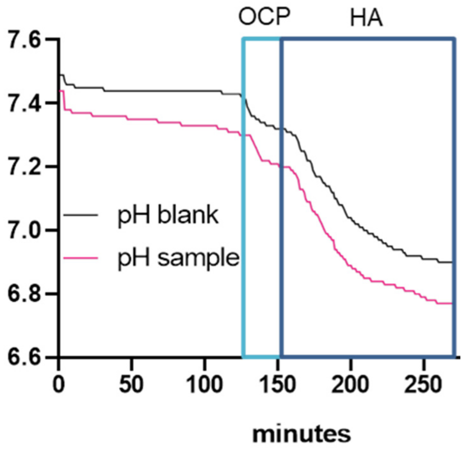

Figure S5. Shows the curves on the pH evolution for the blank (black line) and the sample (pink line): It can be noticed that the presence of the disk does not appear to influence the calcium phosphate formation pathway because the curves have the same shape. The two-step process (i.e., OCP and HA formation) is delimited by both the light blue line and the dark blue line, respectively.
